# The clinical influence of the preoperative lymphocyte‐to‐monocyte ratio on the postoperative outcome of patients with early‐stage gastrointestinal cancer

**DOI:** 10.1002/ags3.12369

**Published:** 2020-07-08

**Authors:** Takayuki Shimizu, Mitsuru Ishizuka, Takayuki Shiraki, Yuhki Sakuraoka, Shozo Mori, Akihito Abe, Yukihiro Iso, Kazutoshi Takagi, Taku Aoki, Keiichi Kubota

**Affiliations:** ^1^ Second Department of Surgery Dokkyo Medical University Tochigi Japan

**Keywords:** immunosuppression, lymphocyte‐to‐monocyte ratio, stage I colorectal cancer, stage I gastric cancer

## Abstract

**Aim:**

The lymphocyte‐to‐monocyte ratio (LMR) is useful for predicting the prognosis of patients with gastric cancer (GC) and those with colorectal cancer (CRC) undergoing surgery. The relationship between the LMR and postoperative outcome of patients with early‐stage gastrointestinal cancers such as stage I GC and CRC remains unclear.

**Methods:**

We retrospectively evaluated 323 stage I GC and 152 stage I CRC patients undergoing surgery. Univariate and multivariate analyses using the Cox proportional hazards model were performed to identify the clinical characteristics associated with overall survival (OS), and the cut‐off values of these variables were determined by receiver operating characteristic analysis. The Kaplan–Meier method and log‐rank test were used for postoperative survival comparisons according to the LMR (GC: LMR < 4.2 vs ≥4.2; CRC: LMR < 3.0 vs ≥3.0).

**Results:**

Univariate and multivariate analyses revealed that OS was significantly associated with the LMR (<4.2/≥4.2) (HR, 2.489; 95% CI, 1.317‐4.702; *P* = 0.005), as well as age (>75/≤75 years) (HR, 3.511; 95% CI, 1.881‐6.551; *P* < 0.001) and albumin level (≤3.5/>3.5 g/dL) (HR, 3.040; 95% CI, 1.575‐5.869; *P* = 0.001), in stage I GC patients. Survival analysis demonstrated a significantly poorer OS in stage I GC patients with a LMR < 4.2 compared with ≥4.2 (*P* < 0.001). In stage I CRC patients, despite a significant difference in OS according to the LMR (<3.0 vs ≥3.0) (*P* = 0.040), univariate analysis revealed no significant association between the LMR and OS.

**Conclusion:**

LMR is a useful predictor of the postoperative outcome of stage I GC patients treated surgically.

## Graphical abstract

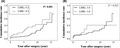

## 1 INTRODUCTION

Although the 5‐year overall survival (OS) rate after surgery in patients with stage I gastric cancer (GC) or colorectal cancer (CRC) is >90%, some patients have poor postoperative outcomes due to recurrence or other diseases.^1^, ^2^ Several studies have revealed that a high age, elevated tumor marker levels, lymphovascular invasion, and male sex are associated with poor postoperative outcomes in patients with stage I GC or CRC.^1^, ^2^ Therefore, predicting postoperative outcomes is important for appropriate postoperative follow‐up of such patients.

During the last decade, many blood‐cell‐based prognostic systems have been reported as useful for predicting the prognosis of GC and CRC patients.^3^, ^4^ For example, the neutrophil‐to‐lymphocyte ratio and platelet‐to‐lymphocyte ratio are blood‐cell‐based prognostic markers for cancer patients. Although the mechanism underlying how these prognostic markers is associated with the prognosis of cancer patients is still unclear, it was previously reported that these markers are associated with cancer‐related inflammation and a tumor microenvironment favoring tumor progression.^5^

Recently, a low peripheral blood lymphocyte‐to‐monocyte ratio (LMR) was reported to be significantly associated with a poor prognosis, including tumor progression and distant metastasis, in patients with GC or CRC.^6^, ^7^, ^8^, ^9^ Additional reports showed that the pretreatment LMR predicts the prognosis of early‐stage cancer patients.^10^, ^11^, ^12^ These findings suggest that the LMR is associated with postoperative outcomes in patients with both stage I GC and CRC. However, the relationship between the LMR and postoperative outcome in patients with early‐stage gastrointestinal cancer remains unclear. Herein, we investigated the relationship between the LMR and postoperative outcomes in both patients with stage I GC and stage I CRC using the database from a single institution.

## 2 METHODS

We retrospectively reviewed 323 stage I GC and 152 stage I CRC patients who underwent surgery between April 2000 and December 2015 at the Second Department of Surgery, Dokkyo Medical University Hospital. We excluded patients with clinical evidence of infection or other inflammatory conditions. All procedures were performed by a single well‐trained surgical team.

This study was approved by the institutional review board (ID number: R‐27‐12J) based on the Ethical Guidelines for Clinical Research of the Ministry of Health, Labour and Welfare in Japan (http://www.mhlw.go.jp/seisakunitsuite/bunya/hokabunya/kenkyujigyou/i‐kenkyu/index.html).

### 2.1 Definition of GC tumor location

Based on the General Rules for Japanese Classification of Gastric Carcinoma (Japanese Gastric Cancer Association, 3rd English Edition), the stomach is anatomically divided into three portions (upper, middle, and lower) delineated by the lines connecting the trisected points on the lesser and greater curvatures. If the tumor involves more than one stomach portion, all involved portions are recorded in descending order of the degree of involvement, e.g., lower, middle or upper, middle, lower.^13^

### 2.2 Statistical analysis

Data are presented as medians with interquartile ranges. Intergroup differences were analyzed using the chi‐squared test or Mann–Whitney *U* test, as appropriate. Clinical factors closely related to OS were identified by univariate and multivariate analyses using the Cox proportional hazards model, with calculation of the hazard ratio (HR) and 95% confidence interval (CI). The Kaplan–Meier method and log‐rank test were used to compare postoperative OS according to the LMR in the GC patients (LMR < 4.2 vs ≥4.2) and CRC patients (LMR < 3.0 vs ≥3.0). All statistical analyses were performed using SPSS software (version 25.0; IBM Co., New York, NY, USA), and differences with a *P*‐value < 0.05 were considered statistically significant.

**Figure 1:**
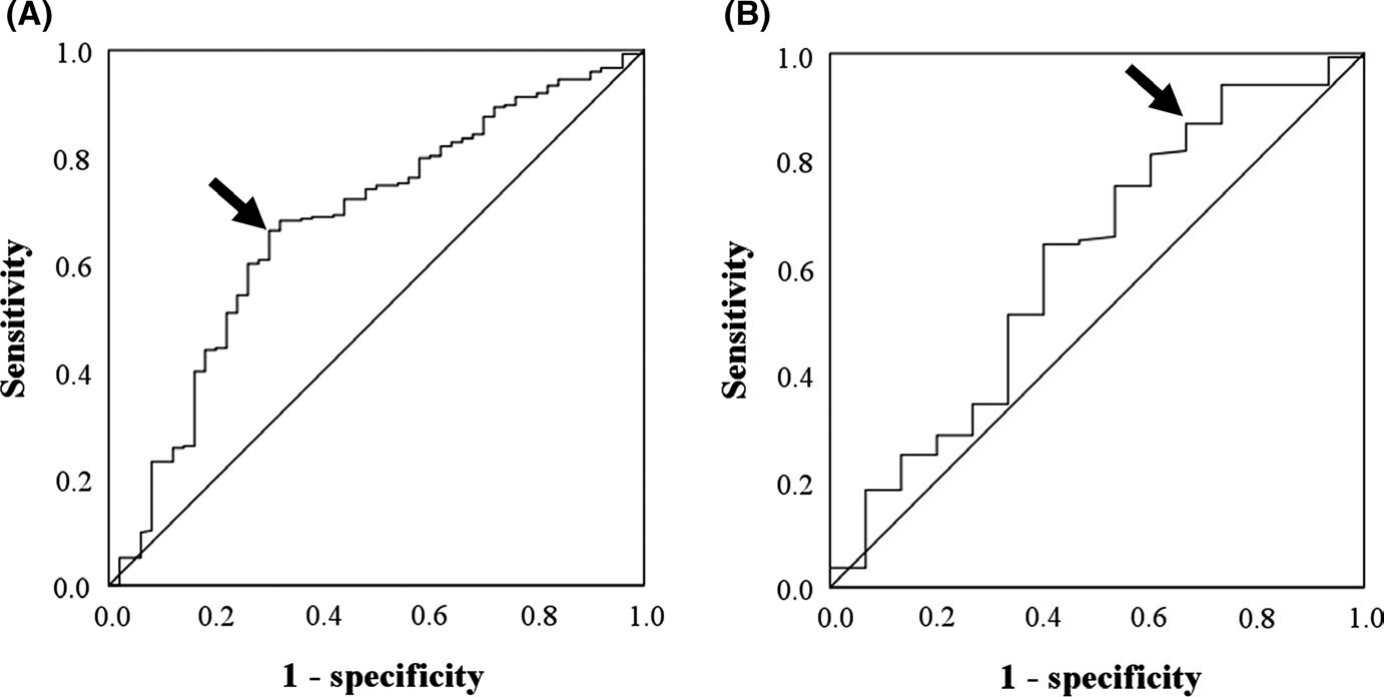
Receiver operating characteristic (ROC) curve showing the optimal cut‐off value of the lymphocyte‐to‐monocyte ratio (LMR). The arrow indicates the most prominent point on the ROC curve. A, Stage I gastric cancer and (B) stage I colorectal cancer

The cut‐off values of the various clinical characteristics evaluated were determined using receiver operating characteristic (ROC) analysis, defined according to the most prominent point on the ROC curve (Youden index = maximum [sensitivity – (1 – specificity)]). We also calculated the area under the ROC curve.^14^ The optimal cut‐off LMR for stage I GC and stage I CRC patients were 4.2 and 3.0, which had sensitivities of 66.3% and 86.9%, specificities of 70.0% and 33.3%, and areas under the ROC curve of 0.673 and 0.610, respectively (Figure 1). Excluding serum levels of carbohydrate antigen 19‐9 (U/mL), carcinoembryonic antigen (ng/mL), and C‐reactive protein (CRP; mg/dL), cut‐off values for other variables, such as age (years), body mass index (kg/m^2^), maximum tumor size (cm), platelet count (×10^4^/mm^3^), serum level of albumin (g/dL), and white blood cell count (×10^3^/mm^3^) were also calculated using ROC analyses.

## 3 RESULTS

### 3.1 Clinical characteristics of stage I GC patients

Of the 323 stage I GC patients (217 males and 106 females) enrolled in this study, 197 had a high LMR (≥4.2) and 126 a low LMR (<4.2). Table 1A shows the clinical characteristics of the stage I GC patients according to the LMR. There were significant differences in age, serum levels of albumin (g/dL), carcinoembryonic antigen (ng/mL) and CRP (mg/dL), sex, number of tumors (1 vs ≥2), survival period (days), and surgery (open vs laparoscopic) according to the LMR. Low LMR (<4.2) was not significantly associated with all comorbidities and the number of comorbidities.

**Table 1:** Relationships between clinical characteristics and LMR in patients with stage I (A) gastric cancer and (B) colorectal cancer

(A)			
Variable	LMR ≥ 4.2 (n = 197) (61.0%)	LMR < 4.2 (n = 126) (39.0%)	*P*‐value
Depth of tumor			
M, SM	177 (54.8%)	111 (34.3%)	
MP	20 (6.2%)	15 (4.7%)	0.621
Gender			
Female	83 (25.7%)	23 (7.1%)	
Male	114 (35.3%)	103 (31.9%)	**<0.001**
Glasgow prognostic score			
0	173 (53.6%)	92 (28.5%)	
1	16 (5.0%)	29 (9.0%)	
2	1 (0.3%)	3 (0.9%)	
Not available	7 (2.1%)	2 (0.6%)	**<0.001**
Location			
EU	0 (0.0%)	1 (0.3%)	
U	33 (10.2%)	27 (8.4%)	
UM	3 (0.9%)	1 (0.3%)	
M	81 (25.2%)	39 (12.1%)	
ML	3 (0.9%)	4 (1.2%)	
L	77 (23.8%)	53 (16.4%)	
Not available	0 (0.0%)	1 (0.3%)	0.265
Lymphatic invasion			
Absence	137 (42.4%)	79 (24.5%)	
Presence	59 (18.3%)	46 (14.2%)	
Not available	1 (0.3%)	1 (0.3%)	0.212
Lymph node metastasis			
N0	187 (57.9%)	120 (37.2%)	
N1	10 (3.1%)	6 (1.8%)	0.899
Number of tumor			
1	180 (55.7%)	105 (32.5%)	
>2	17 (5.3%)	21 (6.5%)	**0.029**
Operation			
Distal gastrectomy	143 (44.3%)	80 (24.8%)	
Proximal gastrectomy	2 (0.6%)	4 (1.2%)	
Total gastrectomy	52 (16.1%)	42 (13.0%)	0.127
Pathological differentiation			
Well or moderately	120 (37.2%)	87 (26.9%)	
Poorly or signet‐ring cell	77 (23.8%)	38 (11.8%)	
Not available	0 (0.0%)	1 (0.3%)	0.113
Surgery			
Open	184 (56.7%)	125 (38.7%)	
Laparoscopic	13 (4.3%)	1 (0.3%)	**0.012**
Venous invasion			
Absence	149 (46.1%)	91 (28.2%)	
Presence	47 (14.6%)	35 (10.8%)	
Not available	1 (0.3%)	0 (0.0%)	0.445
Age (y)	64 (56‐72)	73 (65‐78)	**<0.001**
Albumin (g/dL)	4.0 (3.7‐4.2)	3.8 (3.5‐4.2)	**0.003**
BMI (kg/m^2^)	23.2 (21.1‐25.1)	22.5 (20.5‐25.0)	0.125
CA19‐9 (U/mL)	8.0 (5.7‐14.2)	8.0 (7.0‐17.0)	0.491
CEA (ng/mL)	1.9 (1.4‐3.2)	2.3 (1.7‐3.6)	**0.008**
CRP (mg/dL)	0.1 (0.1‐0.3)	0.3 (0.1‐0.3)	**<0.001**
Maximum tumor size (cm)	2.8 (2.0‐4.0)	3.1 (2.0‐4.4)	0.316
Platelet count (x10^4^/mm^3^)	22.1 (18.2‐25.8)	21.6 (16.7‐24.6)	0.151
Survival period (d)	2056 (1083‐3229)	1629 (593‐2621)	**0.005**
WBC count (×10^3^/mm^3^)	5.8 (4.9‐6.7)	5.6 (4.7‐6.9)	0.848
Diabetes			
Absence	178 (55.1%)	105 (32.5%)	
Presence	19 (5.9%)	21 (6.5%)	0.062
Respiratory disease			
Absence	187 (57.9%)	120 (37.1%)	
Presence	10 (3.1%)	6 (1.9%)	0.899
Cerebrovascular disease			
Absence	191 (59.1%)	119 (36.8%)	
Presence	6 (1.9%)	7 (2.2%)	0.263
Cardiovascular disease			
Absence	173 (53.6%)	116 (35.9%)	
Presence	24 (7.4%)	10 (3.1%)	0.225
Chronic liver disease			
Absence	187 (57.9%)	119 (36.8%)	
Presence	10 (3.1%)	7 (2.2%)	0.851
Renal dysfunction			
Absence	189 (58.5%)	119 (36.8%)	
Presence	8 (2.5%)	7 (2.2%)	0.534
Number of co‐morbidities			
0	139 (43.1%)	87 (26.9%)	
1	42 (13.0%)	22 (6.8%)	
2	13 (4.0%)	15 (4.6%)	
3	3 (0.9%)	2 (0.6%)	0.376

(B)			
Variable	LMR ≥ 3.0 (n = 130) (85.5%)	LMR<3.0 (n = 22) (14.5%)	*P*‐value
Depth of tumor			
M, SM	67 (44.1%)	13 (8.6%)	
MP	63 (41.4%)	9 (5.9%)	0.512
Gender			
Female	74 (48.7%)	19 (12.5%)	
Male	56 (36.8%)	3 (2.0%)	**0.009**
Glasgow prognostic sore			
0	108 (71.0%)	16 (10.5%)	
1	18 (11.9%)	5 (3.3%)	
2	2 (1.3%)	1 (0.7%)	
Not available	2 (1.3%)	0 (0.0%)	0.357
Location			
Colon	89 (58.5%)	16 (10.5%)	
Rectum	41 (27.0%)	6 (4.0%)	0.689
Lymphatic invasion			
Absence	73 (48.0%)	16 (10.5%)	
Presence	54 (35.5%)	6 (4.0%)	
Not available	3 (2.0%)	0 (0.0%)	0.178
Number of tumor			
1	113 (74.3%)	19 (12.5%)	
>2	4 (2.6%)	0 (0.0%)	
Not available	13 (8.6%)	3 (2.0%)	0.413
Pathological differentiation			
Well or moderately	128 (84.2%)	22 (14.5%)	
Poorly	2 (1.3%)	0 (0.0%)	0.558
Surgery			
Open	60 (39.5%)	14 (9.2%)	
Laparoscopic	70 (46.0%)	8 (5.3%)	0.129
Venous invasion			
Absence	70 (47.0%)	10 (6.6%)	
Presence	57 (38.3%)	12 (7.9%)	
Not available	3 (2.0%)	0 (0.0%)	0.401
Age (y)	68 (61‐75)	73 (69‐82)	**0.009**
Albumin (g/dL)	4.1 (3.7‐4.3)	3.9 (3.5‐4.2)	0.154
BMI (kg/m^2^)	23.0 (20.7‐25.3)	23.0 (20.9‐24.8)	0.921
CA19‐9 (U/mL)	7.0 (4.0‐14.2)	7.5 (5.7‐13.2)	0.479
CEA (ng/mL)	2.1 (1.4‐3.4)	2.6 (1.8‐3.6)	0.141
CRP (mg/dL)	0.1 (0.1‐0.2)	0.3 (0.1‐0.6)	**0.007**
Maximum tumor size (cm)	2.5 (1.7‐3.5)	2.0 (1.2‐3.2)	0.207
Platelet count (×10^4^/mm^3^)	22.5 (18.8‐27.0)	20.9 (16.1‐27.8)	0.525
Survival period (d)	1916 (1444‐3127)	1483 (545‐1987)	**0.006**
WBC count (×10^3^/mm^3^)	6.0 (4.6‐7.1)	6.3 (5.6‐6.8)	0.339
Diabetes			
Absence	111 (73.0%)	20 (13.2%)	
Presence	19 (12.5%)	2 (1.3%)	0.487
Respiratory disease			
Absence	127 (83.5%)	18 (11.9%)	
Presence	3 (2.0%)	4 (2.6%)	**0.001**
Cerebrovascular disease			
Absence	120 (78.9%)	21 (13.8%)	
Presence	10 (6.6%)	7 (0.7%)	0.598
Cardiovascular disease			
Absence	110 (72.4%)	14 (9.2%)	
Presence	20 (13.2%)	8 (5.3%)	**0.019**
Chronic liver disease			
Absence	122 (80.3%)	19 (12.5%)	
Presence	8 (5.2%)	3 (2.0%)	0.851
Renal dysfunction			
Absence	121 (79.6%)	21 (13.8%)	
Presence	9 (5.9%)	1 (0.7%)	0.677
Number of co‐morbidities			
0	82 (53.9%)	8 (5.2%)	
1	30 (19.7%)	10 (6.6%)	
2	15 (9.9%)	3 (2.0%)	
3	3 (2.0%)	1 (0.7%)	0.099

Note

Chi‐squared test, Median (IQR), Mann‐Whitney *U* test.

Abbreviations: BMI, body mass index; CA19‐9, carbohydrate antigen 19‐9; CEA, carcinoembryonic antigen; CRP, c‐reactive protein; LMR, lymphocyte‐to‐monocyte ratio; WBC, white blood cell.

### 3.2 Clinical characteristics of stage I CRC patients

Of the 152 stage I CRC patients (93 males and 59 females) enrolled in this study, 130 had a high LMR (≥3.0) and 22 had a low LMR (<3.0). Table 1B shows the clinical characteristics of the stage I CRC patients according to the LMR. There were significant differences in age, serum CRP level (mg/dL), sex and survival period (days) between patients with a high and those with a low LMR. Low LMR (<3.0) was significantly associated with both respiratory disease (*P* = 0.001) and cardiovascular disease (*P* = 0.019).

### 3.3 Postoperative death and recurrence in stage I GC patients

During the observation period, 50 of the GC patients died, including 14 cancer‐related deaths (Table 2A,B). Among the 14 cancer‐related deaths, three patients died of GC, and 11 died of other types of cancers. The other deaths were due to infectious disease in nine patients, cerebrovascular disease in five patients, liver failure in three patients, heart disease, melena, asphyxia, ileus, pulmonary thromboembolism, old age, intra‐abdominal hemorrhage, interstitial pneumonia and a traffic accident in one patient each, and unknown causes in 10 patients. Thirty‐four (27.0%) patients with a LMR < 4.2 compared with 16 (8.1%) patients with a LMR ≥ 4.2 died during the observation period. A LMR < 4.2 was significantly associated with death from an infectious disease (*P* = 0.016). Four patients experienced postoperative recurrence of their GC, of whom two had local recurrence, one had pleural dissemination, and one had lymph node and bone metastases. There were no significant differences in the recurrence pattern according to the LMR in stage I GC patients.

**Table 2:** Relationships between (A) cause of death and (B) recurrence pattern and LMR in patients with stage I gastric cancer

(A)			
Variable	LMR ≥ 4.2 (n = 16)	LMR < 4.2 (n = 34)	*P*‐value
Cerebrovascular disease	1	4	0.058
Gastric cancer	2	1	0.840
Heart disease	0	1	0.210
Infectious disease	2	7	**0.016**
Liver failure	1	2	0.324
Other cancers	5	6	0.282
Other diseases	4	4	0.519
Not available	1	9	**0.001**

(B)			
Variable	LMR ≥ 4.2 (n = 2)	LMR < 4.2 (n = 2)	*P*‐value
Pleural dissemination	1	0	0.423
Local recurrence	0	2	0.076
Lymph node & bone metastases	1	0	0.423

Abbreviation: LMR, lymphocyte‐to‐monocyte ratio.

### 3.4 Postoperative death and recurrence in stage I CRC patients

During the observation period, 15 of the stage I CRC patients died, including five cancer‐related deaths: one from CRC and four from other cancer types (Table 3A,B). Among the non‐cancer‐related deaths, two patients died of infectious disease, and one patient each died of cerebrovascular disease, liver failure, heart disease, hypoglycemia, hypoxemia, old age, hematemesis, and unknown causes. Four (18.2%) patients with a LMR < 3.0 died during the observation period, and a low LMR was significantly associated with death from other cancers (*P* = 0.041) or other diseases (*P* = 0.041). Only 11 (8.5%) patients with a LMR ≥ 3.0 died during the observation period. Two patients had postoperative recurrence of the CRC, one of whom had local recurrence and the other lung metastasis. There were no significant differences in the recurrence pattern according to the LMR in stage I CRC patients.

**Table 3:** Relationships between (A) cause of death and (B) recurrence pattern and LMR in patients with stage I colorectal cancer

(A)			
Variable	LMR ≥ 3.0 (n = 11)	LMR<3.0 (n = 4)	*P*‐value
Cerebrovascular disease	1	0	0.680
Colorectal cancer	1	0	0.680
Heart disease	1	0	0.680
Infectious disease	2	0	0.558
Liver failure	1	0	0.680
Other cancers	2	2	**0.041**
Other diseases	2	2	**0.041**
Not available	1	0	0.680

(B)			
Variable	LMR ≥ 3.0 (n = 2)	LMR<3.0 (n = 0)	*P*‐value
Local recurrence	1	0	0.680
Lung metastasis	1	0	0.680

Abbreviation: LMR, lymphocyte‐to‐monocyte ratio.

### 3.5 Survival of stage I GC patients

**Figure 2:**
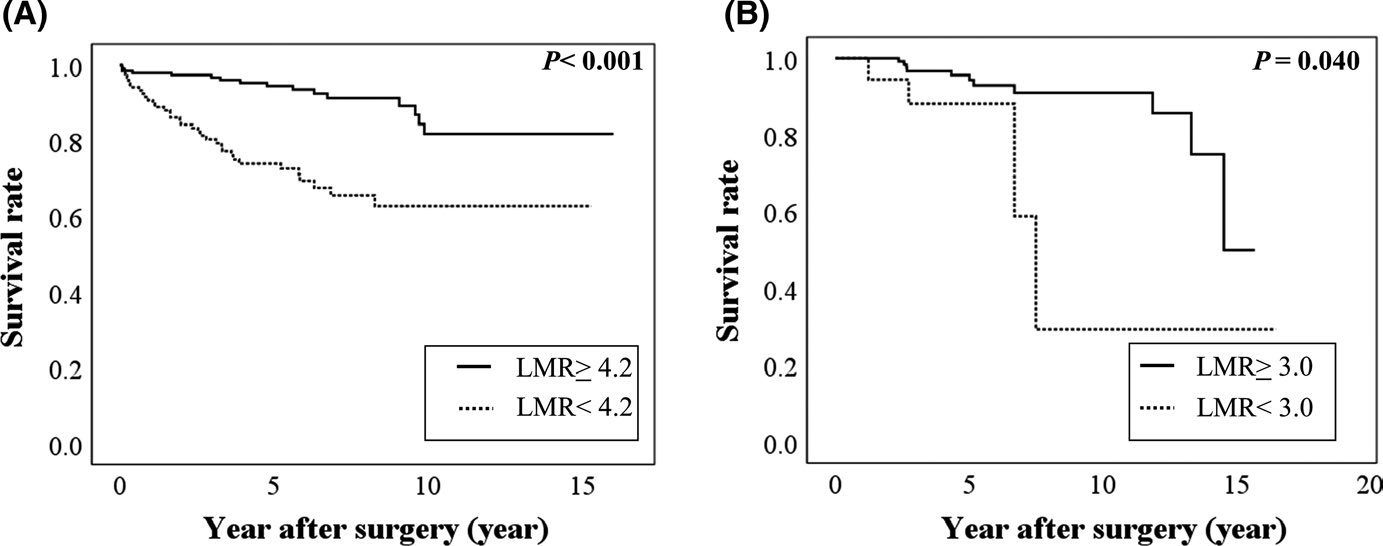
Relationship between overall survival and lymphocyte‐to‐monocyte ratio in patients with early stage gastrointestinal cancer after surgery. A, Stage I gastric cancer and (B) Stage I colorectal cancer

The median and maximum follow‐up periods of the surviving patients with stage I GC were 1905 and 5844 days, respectively, with a mean OS of 2025 ± 1393 days. The Kaplan–Meier method and log‐rank test revealed a significant difference in OS according to the LMR (≥4.2 vs <4.2) (Figure 2A).

### 3.6 Survival of stage I CRC patients

The median and maximum follow‐up periods of the surviving patients with stage I CRC were 1864 and 6009 days, respectively, with a mean OS of 2213 ± 1344 days. The Kaplan–Meier method and log‐rank test revealed a significant difference in OS according to the LMR (≥3.0 vs <3.0) (Figure 2B).

### 3.7 Postoperative incidence of infectious diseases in stage I GC patients

**Figure 3:**
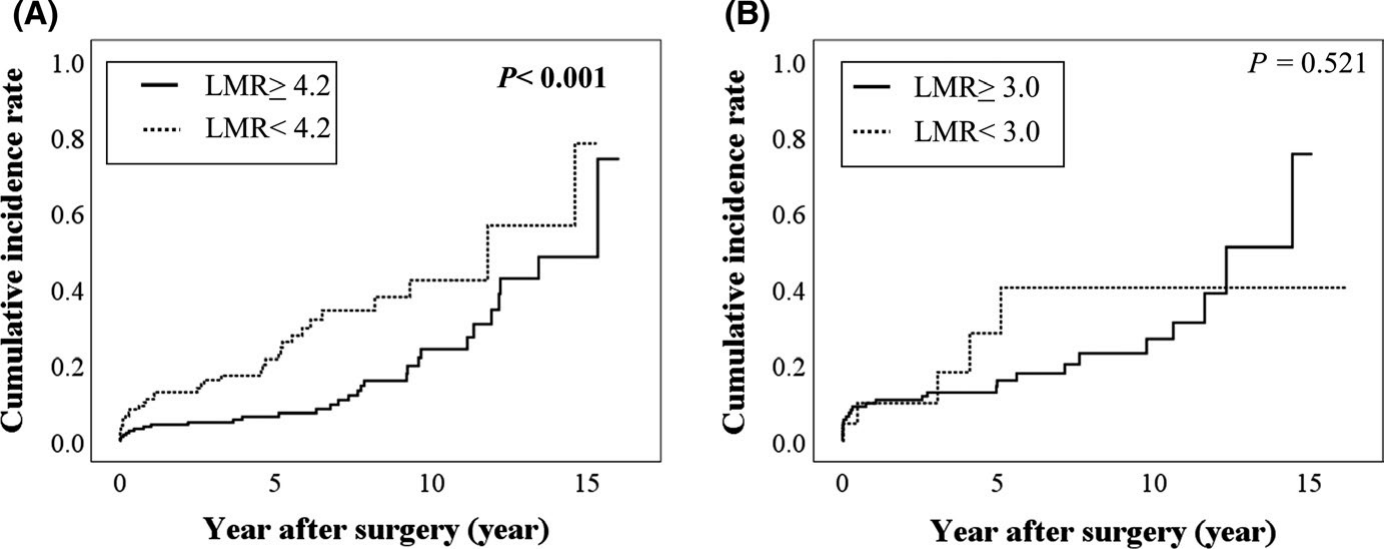
Relationship between cumulative infectious disease and lymphocyte‐to‐monocyte ratio in patients with early stage gastrointestinal cancer after surgery. A, Stage I gastric cancer and (B) Stage I colorectal cancer

During the observation period, 62 GC patients had incidence of infectious diseases. Among the 62 patients, 30 had pneumonia, six had cholecystitis, five had cholangitis, five had shingles, five had skin infection, three had pancreatitis, three had urinary tract infection, one had diverticulitis, one had intra‐abdominal hemorrhage, one had peritonitis, one had spondylitis, and one had sinusitis, respectively. The Kaplan–Meier method and log‐rank test revealed a significant difference between the two groups according to the LMR (≥4.2 vs <4.2) in incidence of infectious diseases (Figure 3A).

### 3.8 Postoperative incidence of infectious diseases in stage I CRC patients

During the observation period, 30 CRC patients had infectious diseases. Among the 30 patients, nine had surgical site infection, nine had pneumonia, six had urinary tract infection, two had enteritis, one had diverticulitis, one had sepsis, one had hepatitis, and one had esophageal candidiasis, respectively. The Kaplan–Meier method and log‐rank test revealed no significant difference between the two groups according to the LMR (≥3.0 vs <3.0) in incidence of infectious diseases (Figure 3B).

### 3.9 Univariate and multivariate analyses of OS in stage I GC patients

Univariate analyses conducted in the stage I GC patients revealed associations of OS with age (>75 vs ≤75 years), serum albumin level (≤ 3.5 vs >3.5 g/dL), body mass index (≤23.0 vs >23.0 kg/m^2^), tumor depth (MP/M or SM), LMR (<4.2 vs ≥4.2), pathological differentiation (poor or signet ring cell vs well or moderate), platelet count (>16.6 × 10^4^ vs ≤16.6 × 10^4^/mm^3^), venous invasion (presence/absence), and white blood cell count (>5.3 × 10^3^ vs ≤5.3 × 10^3^/mm^3^) (Table 4A). These variables were entered into the multivariate analysis, in which a poor OS was significantly associated with the LMR (<4.2/≥4.2) (HR, 2.489; 95% CI, 1.317‐4.702; *P* = 0.005), as well as age (>75/≤75 years) (HR, 3.511; 95% CI, 1.881‐6.551; *P* < 0.001) and serum albumin level (≤3.5/> 3.5 g/dL) (HR, 3.040; 95% CI, 1.575‐5.869; *P* = 0.001) (Table 4A).

**Table 4:** Univariate and multivariate analyses in relation to overall survival of patients with stage I (A) gastric cancer and (B) colorectal cancer

(A)						
Variable	Univariate			Multivariate		
	*P*‐value	HR	95% CI	*P*‐value	HR	95% CI
Age (>75/<75, y)	**<0.001**	5.349	3.021‐9.470	**<.001**	3.492	1.866‐6.535
Albumin (<3.5/>3.5, g/dL)	**<0.001**	3.449	1.856‐6.411	**.015**	13.89	1.677‐115.0
BMI (<23.0/>23.0, kg/m^2^)	**0.023**	1.941	1.094‐3.442	.124	1.623	0.875‐3.009
CA19‐9 (>37/<37, U/mL)	0.317	1.546	0.658‐3.632			
CEA (>5/<5, ng/mL)	0.158	1.726	0.808‐3.683			
CRP (>0.3/<0.3, ng/mL)	0.685	0.826	0.327‐2.083			
Depth of tumor (MP/M or SM)	**0.045**	0.132	0.018‐0.960	.241	0.674	0.028‐1.732
Gender (Male/Female)	0.969	1.012	0.552‐1.856			
Glasgow prognostic score (1 or 2/0)	**0.001**	2.747	1.473‐5.123	.103	0.178	0.022‐1.416
LMR (<4.2/>4.2)	**<0.001**	4.014	2.101‐7.669	**.002**	2.709	1.433‐5.122
Lymphatic invasion (Presence/Absence)	**0.025**	0.452	0.226‐0.905	.564	0.797	0.370‐1.719
Lymph node metastasis (N1/N0)	0.880	1.094	0.340‐3.520			
Maximum tumor size (2.5>/<2.5, cm)	0.105	0.628	0.358‐1.101			
Number of tumors (>2/1)	.066	1.972	0.957‐4.060			
Pathological differentiation (Poorly or signet ring cell/Well or moderately)	**.044**	0.513	0.268‐0.982	.241	0.674	0.348‐1.303
Platelet count (>16.6/<16.6, ×10^4^/mm^3^)	**<0.001**	0.344	0.191‐0.618	.103	0.587	0.309‐1.114
Venous invasion (Presence/Absence)	**0.025**	0.348	0.138‐0.877	.139	0.460	0.165‐1.286
WBC count (>5.3/<5.3, ×10^3^/mm^3^)	**0.003**	0.426	0.241‐0.755	.179	0.653	0.351‐1.215

(B)						
Variable	Univariate			Multivariate		
	*P*‐value	HR	95% CI	*P*‐value	HR	95% CI
Age (>73/<73, y)	0.054	2.717	0.981‐7.524			
Albumin (<3.9/>3.9, g/dL)	**0.006**	5.944	1.658‐21.30	.093	3.346	0.816‐13.72
BMI (<17.5/>17.5, kg/m^2^)	0.050	3.739	1.001‐13.97			
CA19‐9 (>37/<37, U/mL)	0.498	2.037	0.260‐15.95			
CEA (>5/<5, ng/mL)	0.428	1.835	0.409‐8.230			
CRP (>0.3/<0.3, mg/dL)	**0.005**	4.655	1.593‐13.60	.074	2.895	0.903‐9.278
Depth of tumor (MP/ M or SM)	0.804	1.139	0.409‐3.173			
Gender (Male/Female)	0.284	0.534	0.169‐1.684			
Glasgow prognostic score (1 or 2/0)	**0.002**	5.289	1.840‐15.19	.259	2.046	0.591‐7.088
LMR (<3.0/>3.0)	0.051	3.201	0.996‐10.29			
Lymphatic invasion (Presence/Absence)	0.147	2.150	0.764‐6.054			
Maximum tumor size (4.5>/<4.5, cm)	0.205	2.664	0.585‐12.12			
Number of tumors (>2/1)	0.505	0.044	0.000‐435.2			
Pathological differentiation (Poorly/ Well or moderately)	0.841	0.049	0.001‐2.974			
Platelet count (>18.3/<18.3, ×10^4^/mm^3^)	0.464	1.747	0.392‐7.781			
Venous invasion (Presence/Absence)	0.663	1.254	0.454‐3.466			
WBC count (>5.5/<5.5, ×10^3^/mm^3^)	0.644	0.773	0.259‐2.304			

Abbreviations: 95% CI, 95% confidence interval; BMI, body mass index; CA19‐9, carbohydrate antigen 19‐9; CEA, carcinoembryonic antigen; CRP, c‐reactive protein; HR, hazard ratio; LMR, lymphocyte‐to‐monocyte ratio; WBC; white blood cell.

### 3.10 Univariate and multivariate analyses of OS in stage I CRC patients

Univariate analyses among the stage I CRC patients revealed that OS was not significantly associated with the LMR (<3.0/≥3.0), but OS was associated with the serum levels of albumin (≤3.9/>3.9 g/dL) and CRP (>0.3/≤0.3 mg/dL). In the multivariate analysis, a poor OS remained significantly associated with the serum levels of albumin (≤3.9/>3.9 g/dL) (HR, 4.425; 95% CI, 1.215‐16.11; *P* = 0.024) and CRP (>0.3/≤0.3 mg/dL) (HR, 3.691; 95% CI, 1.250‐10.90; *P* = 0.018) (Table 4B).

## 4 DISCUSSION

Consistent with previous studies,^10^, ^11^, ^12^ we found that a low LMR was significantly associated with poor prognosis in patients with early‐stage gastrointestinal cancers (i.e., GC and CRC) (Figure 2). However, very few patients died of GC or CRC (Table 2A, B and Table 3A, B), indicating that the LMR is associated with other causes of death after surgery.

To emphasize the usefulness of LMR in patients with early‐stage gastrointestinal cancer, we compared LMR with the conventional inflammation‐based prognostic score, Glasgow prognostic score (GPS) in prognostication of such patients. Multivariate analyses revealed that GPS was not significantly associated with OS in both stage I GC and stage I CRC patients. A previous study showed that GPS was not good at prognostication of early‐stage cancer patients, because most patients with early‐stage cancer did not have cancer cachexia due to tumor progression.^15^ These facts suggest that LMR is superior to GPS in predicting non‐cancer‐related death after surgery in stage I GC patients.

Our results revealed that a low LMR was significantly associated with older age, hypoalbuminemia, a high serum CRP level, and male sex in patients with early‐stage gastrointestinal cancers (Table 1A,B). Regarding age, the immediate responses to bacterial and viral pathogens are decreased in aged patients because immune responses are affected by aging.^16^ Regarding hypoalbuminemia and high serum CRP levels, recent studies revealed that these characteristics are associated with immunosuppression and malnutrition in cancer patients.^17^, ^18^ All of these findings support that a low LMR reflects immunosuppression due to high age and malnutrition.

A previous study revealed that the LMR was significantly associated with the incidences of infectious diseases, such as pneumonia and urinary tract infections, in patients with acute ischemic stroke.^19^ The authors suggested that the LMR might reflect immunosuppression induced by stroke, and in turn, the immunosuppression is the cause of infectious diseases.^19^ Therefore, the LMR is useful for predicting the outcome of not only patients with cancer but also those with heart or vascular disease.^20^, ^21^ Thus, immunosuppression might be the underlying cause of the deaths attributed to other diseases in our patients with a low LMR.

In fact, our results showed that a low LMR was significantly associated with death from infectious diseases among the stage I GC patients (*P* = 0.016) (Table 2A,B) and with death from other cancers (*P* = 0.041) and other diseases (*P* = 0.041) among the stage I CRC patients (Table 3A,B). These findings support that a low LMR might be useful for predicting death from a wide variety of diseases, including infectious diseases, in patients with early‐stage gastrointestinal cancers after surgery.

Although the survival analysis indicated a poorer OS in the stage I CRC patients with a low LMR (<4.2), multivariate analysis did not identify a significant association between the LMR and OS in these patients. There was a difference in the distributions of stage I GC and stage I CRC patients according to the LMR, in that the patients with a low LMR comprised 39.0% (126/323) of the total GC cohort compared with 14.5% (22/152) of the CRC cohort. Because the proportion of patients with a low LMR was higher among stage I GC patients than stage I CRC patients, there might have been a difference between the two groups in the multivariate analyses.

The preoperative LMR might be useful for prognostication in stage I GC patients, because GC is associated with postoperative weight loss. Unlike in CRC patients, gastric storage dysfunction, reduced ghrelin levels, and digestion/absorption disorders lead to postoperative weight loss in GC patients.^22^, ^23^, ^24^ According to recent studies, being underweight is associated with increased incidences of stroke, atrial fibrosis, and impaired endothelial dysfunction,^25^, ^26^, ^27^ as well as an increased risk of pneumonia.^28^ Thus, the combination of postoperative weight loss and a low LMR might increase the risks of other diseases, leading to a worse postoperative outcome in stage I GC patients.

Recent studies showed that oral nutritional supplements significantly improved postoperative weight loss in GC patients.^29^, ^30^ In the same way, another study showed that exercise interventions prevented postoperative muscle loss in GC patients.^31^ In addition, exercise interventions prevented not only incidence of cancer and cardiovascular disease but also all‐causes of mortality.^32^, ^33^ Therefore, in order to prevent non‐cancer‐related death, both nutritional supplements and exercise interventions would be needed in GC patients with low LMR (<4.2).

There were some limitations to our study. First, this was a retrospective study conducted at a single institution. Second, the population of stage I CRC patients in this study was relatively small (n = 152). To overcome these limitations, validation of our results in multi‐institutional studies with larger sample sizes is needed.

In conclusion, the present findings indicated a relationship between the preoperative LMR and the outcome of patients with early‐stage gastrointestinal cancer. The novelty of the study is that LMR could predict not only primary cancer death but also non‐cancer‐related death due to infectious and vascular diseases. Based on these results, the LMR could be considered a factor determining both nutritional supplements and exercise interventions for such patients.

## DISCLOSURE

Conflicts of Interest: The authors declare no conflicts of interest regarding the publication of this paper.
